# The World Hip Trauma Evaluation (WHiTE) platform trial: a framework for randomized comparisons of interventions for fragility hip fracture

**DOI:** 10.1302/2633-1462.64.BJO-2024-0240

**Published:** 2025-04-02

**Authors:** Matthew L. Costa, Juul Achten, Duncan Appelbe, Amrita Athwal, Richard Grant, Jonathan Cook, Rafael Pinedo-Villanueva, Stavros Petrou, Xavier L. Griffin

**Affiliations:** 1 Oxford Trauma and Emergency Care, Nuffield Department of Orthopaedics, Rheumatology and Musculoskeletal Sciences, University of Oxford, Oxford, UK; 2 UK Musculoskeletal Trauma PPI Group, Nuffield Department of Orthopaedics, Rheumatology and Musculoskeletal Sciences, University of Oxford, Oxford, UK; 3 Centre for Statistics in Medicine, Nuffield Department of Orthopaedics, Rheumatology and Musculoskeletal Sciences, University of Oxford, Oxford, UK; 4 Nuffield Department of Primary Care Health Sciences, University of Oxford, Oxford, UK; 5 Barts Bone and Joint Health, Blizard Institute, Queen Mary University London, London, UK

**Keywords:** Hip, Fracture, Platform, Trial, hip fractures, Trauma, EQ-5D-5L, EQ-5D, clinical studies, hip fracture surgery, patient-reported outcome measure, randomized clinical trials, statistical analysis

## Abstract

**Aims:**

Hip fracture is one of the biggest challenges facing patients and healthcare systems. Worldwide, there are currently 1.3 million hip fractures per year, projected to rise to more than six million by 2050. This protocol describes a platform trial framework, designed to efficiently deliver multiple randomized comparisons of interventions for patients with a fragility hip fracture.

**Methods:**

All patients aged 60 years and over with a hip fracture presenting to the World Hip Trauma Evaluation (WHiTE) recruitment centres will be considered for eligibility for each of the randomized comparisons appended to the platform at the time of recruitment. They will be offered the opportunity to take part in any or all of the randomized comparisons for which they are eligible. Comparisons may be contemporaneous or distributed throughout the treatment pathway. This master protocol describes the trial procedures, core dataset, and documentation. It describes those components of the research process which will be consistent between randomized comparisons. Where additional procedures are planned, specific to a randomized comparison, these will be described in a separate appendix protocol for that randomized comparison.

**Conclusion:**

The WHiTE platform trial will provide randomized evidence regarding the clinical and cost-effectiveness of interventions to improve outcomes for patients with fragility hip fracture. Findings will inform national and international policy and practice guidelines for the management of patients with a hip fracture.

Cite this article: *Bone Jt Open* 2025;6(4):383–390.

## Introduction

Hip fracture is one of the biggest challenges facing patients and healthcare systems. Worldwide there are 1.3million hip fractures with more than 70,000 hip fractures in the UK every year.^1^ These figures are projected to double by 2060.^2^ The global cost of this clinical problem is estimated at 1.75 million disability-adjusted life years lost, and represents 1.4% of the total healthcare burden in established market economies.^3,4^ People suffering hip fracture have a 30-day mortality rate of 7%, a one-year mortality rate of 25%, and experience a permanent reduction in their health-related quality of life similar to that of a patient with Parkinson’s disease or multiple sclerosis.^5^

The World Hip Trauma Evaluation (WHiTE) cohort study,^5-15^ and embedded randomized trials,^16-23^ has been delivering high-quality evidence to inform the care of patients with fragility hip fracture for the last ten years. Using the findings from these studies, we describe the new WHiTE platform trial, designed to efficiently deliver multiple, simultaneous, randomized comparisons of interventions for patients aged 60 years and over with a hip fracture. The purpose of this platform is to simplify the patient pathway through research in this field, and leverage efficiencies in the reduction of required documents and alignment of data collection. The platform benefits from a coherent and consistent single set of ethical and regulatory approvals, and an explicit legal basis and processing purpose for the use of patient-level deidentified, personal data.

This master protocol describes those components of the research process which will be consistent between randomized comparisons. Where additional procedures are planned, specific to a randomized comparison – for example, the collection of additional outcome data – these will be described in a protocol appendix for that randomized comparison. Each randomized comparison will have its unique start and stop dates and publication of results, and will be conducted in a way that does not compromise the integrity of the platform and other concurrent randomized comparisons.

## Methods

This project was developed by a team of patient representatives from the UK Musculoskeletal Trauma Patient and Public Involvement Group, clinical experts in the care of patients with hip fracture, trial management specialists, and experienced statisticians and health economists.

### Trial design

The WHiTE platform trial provides an overarching framework, designed to efficiently deliver multiple randomized comparisons of interventions for older people with a hip fracture. Details of each comparison will be described in a protocol appendix, and regulatory approvals will be obtained through the substantial amendment process.

All patients aged 60 years and over with a hip fracture presenting to the WHiTE recruitment centres will be considered for eligibility for each of the randomized comparisons running at that centre within the platform at the time of recruitment. They will be offered the opportunity to take part in any or all of the randomized comparisons for which they are eligible. Within a randomized comparison, the aspect of care being assessed may relate to different elements of the treatment pathway.

**Fig. 1:**
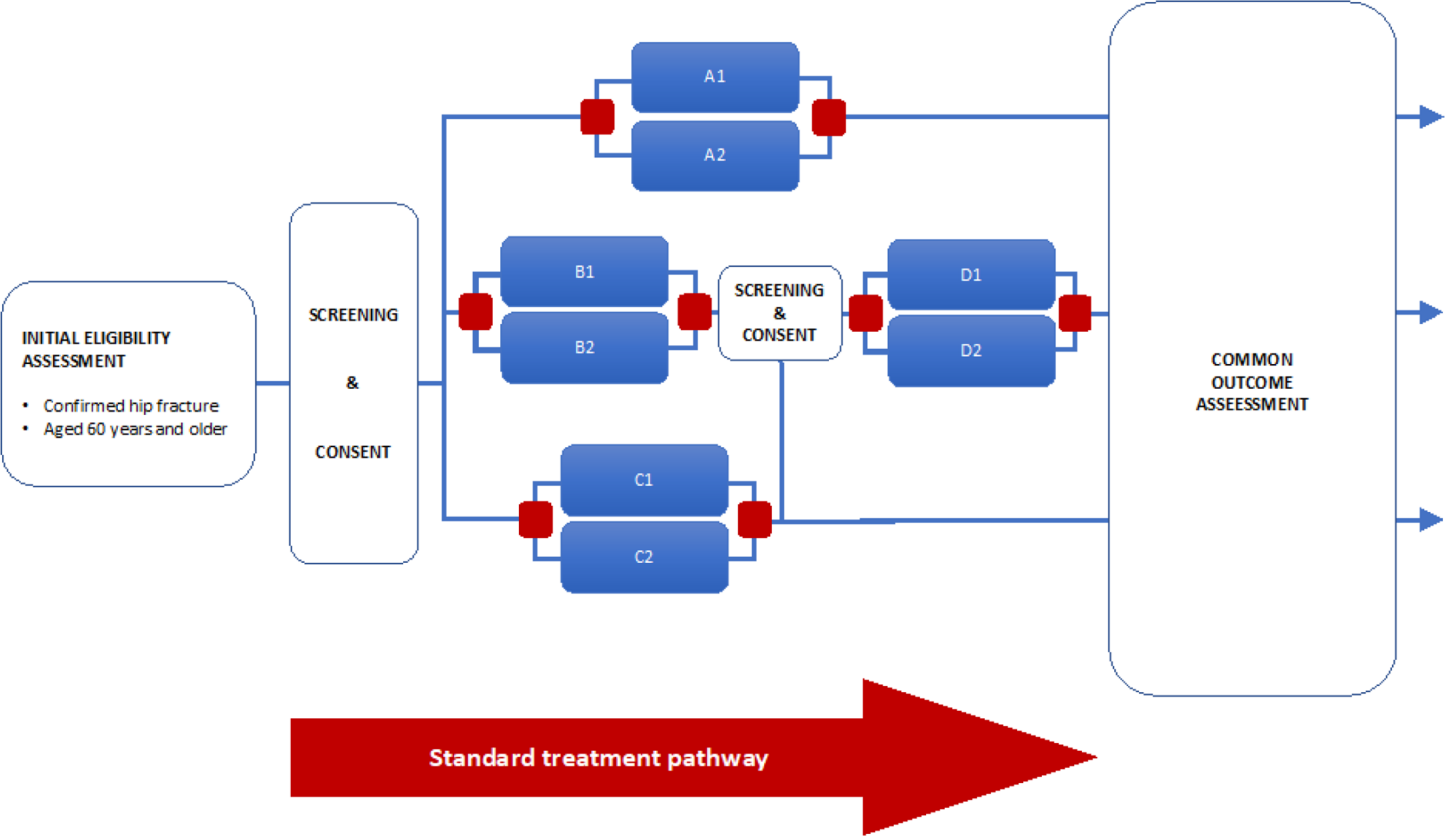
World Hip Trauma Evaluation (WHiTE) platform summary. Key: 1/2 are randomly assigned treatment alternatives for clinical interventions A, B, C, and D.

Eligibility for each randomized comparison will be assessed against the specific criteria described in the relevant protocol appendix. Interventions may be simple, complex, or multimodal; for example, clinical trial of investigational medicinal products, surgical interventions, or care pathways; delivered at any stage along the diagnostic, treatment, and rehabilitation pathway. Figure 1 provides an illustration of the flow through the platform with four hypothetical comparisons (A to D).

### Recruitment centres

Each recruitment centre will routinely provide care for patients with hip fracture. Each centre has a written standardized care pathway for hip fracture patients, a named lead clinician, appropriately trained research staff, appropriate capacity for data collection, and a willingness to screen all eligible patients. Centres that agree to take part in the platform will not be obliged to participate in all randomized comparisons.

### Participants

All adults aged 60 years or over diagnosed with a hip fracture by the treating clinical team at the recruitment centres will be potentially eligible for the platform. If deemed eligible for the platform, they will be assessed against comparison-specific eligibility criteria as described in the relevant comparison protocol.

### Inclusion criteria

- Adults aged 60 years and over with a hip fracture.

### Exclusion criteria

- Previous participation in the same randomized comparison.
- A second hip fracture (contralateral side) while the patient is still enrolled in the platform following their first hip fracture. Enrolment for a second time to the WHiTE platform based on a second fracture is possible once all final follow-up timepoints for the comparisons in which a participant is already enrolled have been completed.

### Screening and eligibility assessment

A member of the clinical team, with routine access to the patient’s personal data, will screen each patient to determine their age and diagnosis of a hip fracture. All potentially eligible patients will be screened and assessed for eligibility for entry into each randomized comparison by a member of staff delegated to conduct screening.

### Informed consent

Once eligibility for any of the randomized comparisons has been confirmed, informed consent will be sought. For those participants who are eligible for further randomized comparisons later in the treatment pathway, additional consent discussions will be undertaken as appropriate.

Patients will be presumed to have capacity unless established otherwise, and the default will be to seek prospective individual consent from every patient. However, patients with a hip fracture are a clinical priority for urgent operative care, are in pain, and have often received opiate analgesia. It is therefore understandable that the majority of patients find the initial period of their treatment confusing and disorientating. Similarly, patients’ next of kin, carers, and friends are often anxious at this time, and may have difficulty in weighing the large amounts of information that they are given about the injury and plan for treatment. The clinical team will make a judgement about the amount and complexity of the information that the participant is able to understand and retain on an individual basis, and whether individuals have capacity to consent or whether to approach a personal or professional contact on their behalf. For guidance on the assessment of capacity for comparisons that do not involve investigational medicinal products, recruitment centres in England and Wales will refer to guidance from the Mental Capacity Act 2005 to assess the patient’s decision-making capacity;^24^ those in Scotland will refer to that of the Adults with Incapacity (Scotland) Act 2000;^25^ and those in Northern Ireland to the Mental Capacity Act 2016 (Northern Ireland).^26^ For comparisons that include investigational medicinal products, recruitment centres will refer to the Medicines for Human Use (Clinical Trials) Regulations for guidance.^27^

### Randomization

All centres will have access to an electronic device with web access to a secure, 24-hour, web-based randomization system. When a patient is eligible for participation in a randomized comparison and consent has been obtained, sufficient identifiable details will be logged on a secure, encrypted, web-based system. Basic information including the participant initials, date of birth, and eligibility checks will be entered. The participant will then receive a unique randomized comparison-specific ID; they will have one per randomized comparison they are participating in, in addition to an overall platform-specific ID. The allocation sequence(s) for each randomized comparison will be generated as described in detail in the relevant protocol appendix, and will be carried out independently of any other concurrent randomized comparison.

### Participant withdrawal

Participants may decline to continue to take part in the platform, either from individual comparisons if they are recruited to multiple, or from the whole platform if they want to withdraw from it, at any time without prejudice. A decision to decline consent or withdraw will not affect the standard of care the patient receives.

### Outcomes

Baseline data: after a patient is enrolled, a member of the local research team will approach the participant with a questionnaire including a (retrospective) assessment of pre-injury generic health-related quality of life using the EuroQol five-dimension five-level (EQ-5D-5L) questionnaire,^28^ as well as questions about pre-injury resource use, residential and mobility status, and relevant medical history. Hospital data regarding admission assessment and treatment, where appropriate, will be collected.

Outcome data: we will collect a common outcome dataset (Table I) across all randomized comparisons at four months after diagnosis of a hip fracture as a minimum. In addition, longer-term outcomes will be collected using routinely collected data up until the last follow-up timepoint for the participant according to the randomized comparison(s) in which they are enrolled.

**Table I.** Common outcome dataset for the WHiTE platform.

Outcomes	Objectives	Outcome measures	Timepoint(s)
Short-term outcomes	To compare HRQoL between treatment groups	EQ-5D-5L	Baseline and at four months post-diagnosis of a hip fracture
	To compare mobility between treatment groups	mNMS	Baseline and at four months post-diagnosis of a hip fracture
	To compare residential status between treatment groups	UK National Hip Fracture Database Residential Status	Baseline and at four months post-diagnosis of a hip fracture
	To compare mortality risk between treatment groups	Death notification	Up to four months post-diagnosis of a hip fracture
	To compare risk and pattern of complications between treatment groups	Complications CRF, medical records check	Baseline and at four months post-diagnosis of a hip fracture
	To compare the healthcare and broader resource implications between treatment groups	Review of hospital medical notes complemented by patient-completed resource use questionnaire	Baseline and at four months post-diagnosis of a hip fracture
Long-term outcomes	To compare risk and pattern of complications between treatment groups	Bespoke diagnostic and procedural events within linked routinely collected databases	Up to final appendix-specific follow-up timepoint
	To compare the healthcare and broader resource implications between treatment groups	Bespoke diagnostic and procedural events and healthcare contact reimbursement data within linked routinely collected databases	Up to final appendix-specific follow-up timepoint
	To compare mortality risk between treatment groups	Linked routinely collected registers of death events and attributed causes	Up to final appendix-specific follow-up timepoint

CRF, clinical reporting form; EQ-5D-5L, EuroQol five-dimension five-level questionnaire; HRQoL, health-related quality of life; mNMS, modified New Mobility Score.

Any other outcome collection will be described in full in the relevant protocol appendix depending upon the nature of the randomized comparison(s) in which the participant has enrolled. Additionally, for comparisons including investigational medicinal products, depending on the risk and status of the medicinal product, part of the objectives will be to collect safety endpoints, as determined by the risk assessment of that interventional arm.

### Health-related quality-of-life

The primary outcome measure is the EQ-5D-5L index at four months post-diagnosis of a hip fracture.^28^ The EQ-5D-5L is a validated measure of health-related quality of life, consisting of a five-dimension (mobility, self-care, usual activities, pain/discomfort, anxiety/depression) health status classification system and a separate visual analogue scale (VAS).^29^ Parsons et al^20,30^ demonstrated that the EQ-5D correlated strongly with a hip-specific patient-reported outcome measure (Oxford Hip Score);^31,32^ it has an independently determined minimal clinically important difference for hip fracture surgery; and can be completed by patient proxies such as relatives when the patients are unable to self-report. Health status plateaus four months after hip fracture surgery, and this is the timepoint when this measure is collected for the National Hip Fracture Database (NHFD). Assessing EQ-5D outcomes provides consistency with other clinical studies in this patient population.^15,30^ The EQ-5D is the recommended instrument in the UK core outcome set for hip fracture.^33^

EQ-5D-5L summary index values will be derived using the most up-to-date guidance from the National Institute for Health and Care Excellence (NICE);^34^ currently, this recommends mapping EQ-5D-5L descriptive system data onto the EQ-5D-3L valuation set using the Crosswalk Index Value Calculator.^35^ The scale for this value set ranges from -0.594, indicating the worst possible heath state, to 1.0, and is anchored at 0 and 1.0 where these values indicate health states equivalent to death and full health, respectively. The EQ-5D is NICE’s preferred measure of health-related quality of life, and it is used to value health effects in the assessment of health interventions. ^36^

Using an anchor point for death, EQ-5D can be imputed for participants who do not survive to the primary timepoint of four months, which is particularly relevant in this population. Parsons et al^14^ modelled patient EQ-5D recovery trajectories after hip fracture surgery to assess the extent of any bias in four-month outcomes by comparing complete case analysis, model-based projections, and data imputation. They showed that imputing a utility value of zero for death resulted in a very close approximation to the more complex projection methods, which were highly dependent on early (pre four-month) EQ-5D score data that would not be available in the setting of a trial.^14^ The EQ-VAS will also be collected as part of the EQ-5D-5L questionnaire.

### Mortality

Qualitative work with patients who sustain hip fractures identified mortality as an important metric.^33^ This will be recorded by recruitment centres at discharge from the medical records, or at any point during follow-up. These data will be confirmed through linkages with Civil Registration (Deaths) (England & Wales), the General Register Office for Northern Ireland, and the Statutory Registers of Births, Deaths and Marriages in Scotland.

### Subjective walking performance

The modified New Mobility Score (mNMS)^37^ is a multicomponent instrument that was developed originally to measure mobility in older adults with hip fracture in post-acute and community settings.^38^ The instrument assesses ambulation inside the home, outside the home, and while shopping. A score of zero to three points is given for each component, resulting in a total score of zero to nine points.

### Residential status

Changes in residential status provide a marker for the patients’ independence through their hip fracture recovery and is one of the recommended core outcomes for trials assessing interventions in hip fractures.^33^ It will be reported by participants or their proxy using an ordinal scale as per the NHFD: 1) own home/sheltered housing; 2) residential care; 3) nursing care; 4) rehabilitation unit – hospital bed in the current trust; 5) rehabilitation unit – hospital bed in another trust; 6) rehabilitation unit – NHS-funded care home bed; and 7) acute hospital.

### Complications

All expected serious adverse events (SAEs) related to the fracture, standard surgical procedure, or the randomized non-investigational medicinal product (IMP) comparisons will be recorded as complications, unless they are more severe than expected, in which case they will be reportable SAEs. These events will be reported by recruitment centres as they become aware of events, as well as by participants, carers, or consultees.

### Resource use

Clinical reporting forms will be designed to collect information on use of resources from medical records at the treating hospital during the initial inpatient stay. Further resource use data will be collected from the participants to complement the medical records. Data collected will include hospital contacts related to the index fracture with hospitals other than the index recruitment centre, rehabilitation units, and other care settings. Questions will also be asked about community health and social care resource use, use of equipment, and changes to the home, such as bath rails, related to the index fracture. To estimate burden on families, questions will be asked about private expenses with rehabilitation services, informal care, and loss of productivity.

Resources required to deliver the different types of treatment will be valued by liaising with local finance departments to review tariffs and healthcare resource groups. Further health and social care will be valued using national unit cost estimates for health and social care resource inputs from the Department of Health and Social Care when available.^39^ Curtis and Burns^39^ also include unit estimates for equipment and home changes. Informal care, productivity losses, and lost income will be valued using age- and sex-specific Office of National Statistics or equivalent weekly average earnings estimates following a human capital approach. In sensitivity analyses, assumptions will be varied to estimate robustness of results to different costing approaches.

### Data linkage for routinely collected patient-level data

Individual participant consent will be obtained for two separate groups of linkages. First, participants will be asked for consent to access their patient-level routinely collected data captured by the various UK data warehouses that hold information, including death information, and diagnostic and procedural codes relevant to hospitalizations and/or outpatient attendances for patients treated in NHS hospitals. These will provide a measure of long-term outcomes and NHS resource use and mortality. Second, participants will also be asked for consent to access patient-level routinely collected data captured by the two ongoing national hip fracture audits in the UK.

### Safety reporting

In order to make the safety reporting schedules for the WHiTE platform efficient, those requirements applied to clinical trials of investigational medicial product (CTIMP) comparisons will be generalized across the entire platform.

Across all of the comparisons, SAEs which are related to and expected in the course of a hip fracture before, during, and after the admission for a hip fracture, including standard surgical procedures, will be exempt from reporting as SAEs across all comparisons unless the event is considered related to an IMP intervention. Instead, all other events will be reported as a complication.

The events that are exempt from reporting as SAEs will be classified as general or surgery-specific complications.

## Statistics

### Summary of the general statistical approach

A fully detailed statistical analysis plan (SAP) will be prepared for each randomized comparison and finalized after review by the independent Project Oversight Committee. A summary of the statistical approach and methods for the common outcome dataset across the platform is provided here.

Principal analyses will be based on the intention to treat (ITT) principle (i.e. participants with available data will be analyzed as they were randomized regardless of treatment received). Further analyses of different populations and targeting different estimands (e.g. per-protocol or as treatment) may be undertaken as outlined in the relevant randomized comparison protocol appendix.

Baseline demographic data will be summarized to check comparability between treatment arms. Standard statistical summaries and graphical plots will be used to present findings for the primary outcome measure and secondary outcome measures. The principal analyses will be supplemented where appropriate with sensitivity analyses. The main analytical method is expected to be mixed-effects models, and analyses will adjust for stratification factors and important baseline covariates to maximize precision for EQ-5D-5L and other continuous longitudinal outcomes. Details of adjustment will be pre-specified in the relevant randomized comparison protocol appendix and SAP. With regard to the common outcome set, the EQ-5D-5L index score at four months will be analyzed by calculating an adjusted treatment effect by using a mixed-effects linear model to compare the EQ-5D-5L score at four months (with a zero value imputed for those who have died at this timepoint) between the treatment arms adjusting for stratification factors (as per the relevant randomized comparison, e.g. age, sex, and cognitive impairment) as fixed effects, and including recruitment centre as a random effect (or using cluster robust variance) to allow for heterogeneity in the response between recruitment centres. A sensitivity analysis of EQ-5D-5L at four months with additional adjustment for the retrospective pre-injury baseline EQ-5D-5L will be performed to enable the influence of this factor to be evaluated. Other sensitivity analyses will be fully described in the SAP for the relevant randomized comparison. Common outcomes will be similarly analyzed as far as possible, with logistic regression being used for binary data and linear regression for continuous data.

Complications and other adverse events will be summarized, and comparisons will be considered exploratory unless otherwise indicated within the specific comparison documentation (e.g. randomized comparison protocol appendix and statistical analysis plan).

Each set of randomized comparison analyses conducted within the WHiTE platform will be evaluated separately in terms of statistical significance. The statistical significance will be assessed at 5% for two-sided tests and reported for p-values less than 5% (p < 0.05).

### Summary of the general health economic approach

A fully detailed health economics analysis plan (HEAP) will be prepared for each randomized comparison and finalized after review by the independent Project Oversight Committee. A summary of the core economic evaluation approaches for the common outcome dataset across the platform is provided here.

The economic evaluation will express cost-effectiveness in terms of incremental cost per quality-adjusted life year (QALY) gained associated with the experimental intervention from a health service and personal social services perspective at four months post-diagnosis. We will report health and social care resource use values and their associated economic costs between diagnosis and four months post-diagnosis, using data extracted from bespoke resource use CRFs and participant-completed questionnaires designed for each randomized comparison. Fractures in this elderly population may burden their carers, and it is possible that different treatment pathways will have different consequences on their families and friends. As such, we will also report separately private expenses, informal care, and productivity losses incurred in both groups for patients and carers.

Any missing costs and QALYs will be jointly imputed using multiple imputation chained equations. Cost and QALY estimates will be bootstrapped and adjusted for stratification variables (e.g. recruitment centre) and other potential variables as per the SAP, such as age, sex, and cognitive impairment, in secondary analyses. ‘All available’ and ‘imputed’ cost categories and QALY data will be reported by treatment group in a cost-consequences framework. The key cost-effectiveness parameter will be the bootstrapped incremental net monetary benefit statistic (INMB) derived using the recommended UK societal cost-effectiveness thresholds of £20,000 and £30,000 per QALY.^40^ The INMB estimates the added benefit of the intervention, if any, by subtracting its extra cost from the economic value of the additional benefits, as indicated by the cost-effectiveness threshold. Positive INMB values indicate the intervention is cost-effective. Using cost-effectiveness acceptability curves, we will depict the probability of the interventions being cost-effective at a range of cost-effectiveness thresholds to illustrate the uncertainty around the adoption decision. In one-way sensitivity analyses and scenario analyses, we will vary methodological assumptions to gauge robustness of results.

### Data management

The Oxford Clinical Trials Research Unit (OCTRU) at the University of Oxford will facilitate the platform data collection system containing demographic and outcome data for each of the participants. Personal data collected via the platform will be handled and stored in accordance with the University of Oxford data protection policies, as well as the General Data Protection Regulation and Data Protection Act 2018, which require data to be deidentified as soon as it is practical to do so.

To ensure compliance with regulations, direct access will be granted to authorized representatives from the sponsor, host institution, and the regulatory authorities to permit relevant monitoring, audits, and inspections.

For the purpose of analyses, the research team will only process deidentified patient-level data. Data required as evidence for publications will be appropriately processed including deidentification and suppression of fields with low data counts. Where possible, aggregated (rather than individual) data will be supplied.

### Quality control

Quality control procedures will be undertaken during the recruitment and data collection phases of each randomized comparison to ensure research is conducted, generated, recorded, and reported in compliance with the master protocol and relevant appendices, good clinical practice (GCP), and ethics committee recommendations. The Lead Investigators and Comparison Managers will develop all data management and monitoring plans, and a risk-adapted approach will be taken for each comparison to ensure the appropriate level of monitoring takes place by the central research team (OCTRU).

### Oversight committees

The day-to-day management of each randomized comparison will be overseen by the Comparison Management Groups (CMG), who will meet monthly to assess progress. Each comparison will have its own Comparison Manager, who will be responsible for the training of research staff at each of the recruiting centres for that comparison. The core Platform Management Group (PMG) consists of the senior members of staff involved in the design, set-up, and management of the platform, and will oversee all of the randomized comparisons.

The independent Platform Oversight Committee (POC) provides overall supervision of the platform. Its terms of reference will be agreed within a POC charter, which will outline its roles and responsibilities. Meetings of the POC will take place at least once a year when there are randomized comparisons open to recruitment, and they will review the progress of each active comparison at that time. The independent Data and Safety Monitoring Committee (DSMC) is a group of experts external to the platform who assess the progress, conduct, participant safety, and, if required, critical endpoints of the platform and the appended comparisons. The platform DSMC will adopt a DAMOCLES-based charter,^41^ which defines its terms of reference and operation in relation to oversight of the platform.

### Dissemination

Outputs from the WHiTE platform will be prepared for each randomized comparison independently. The dissemination strategy will consist of three strands.

Our patient representatives will lead dissemination to the patients and carers directly through their extensive network of patient advocacy organizations, including the Royal Osteoporosis Society. They will help generate plain language summaries for patients and the public.

To reach the clinical community, we will produce free-to-access publications in the mainstream literature, and submit for presentations at national and international multidisciplinary meetings including the Global Fragility Fracture Network (FFN) Congress.

In addition, we are developing complementary systems incorporating non-traditional media, such as podcasts and animated videos, to disseminate results to the wider public.

## Discussion

Recent international cohort and registry observational studies have demonstrated that clinical practice remains variable worldwide for patients with hip fractures.^42^ This variation is present through the initial assessment of patients, surgical and perioperative care, rehabilitation, and secondary prevention of future fractures. There is a pressing need to extend and strengthen the evidence base throughout the pathway of care.

The WHiTE platform trial offers the opportunity to test multiple interventions to improve patient outcomes throughout the care pathway using an established network of recruiting centres and efficient design methodology.

**Take home message**

- The evidence base informing the management of patients with a hip fracture has changed dramatically in recent years, but there remain many unanswered research questions.

- While randomized clinical trials remain the gold standard for assessing new interventions, traditional trial designs have been criticized for being inefficient, taking a long time, and therefore being expensive.

- The WHiTE platform trial is designed to address these criticisms, while also making it easier for patients to take part in hip fracture research.

## Data Availability

The datasets generated and analyzed in the current study are not publicly available due to data protection regulations. Access to data is limited to the researchers who have obtained permission for data processing. Further inquiries can be made to the corresponding author.
